# Intramyocardial implantation of differentiated rat bone marrow mesenchymal stem cells enhanced by TGF-β1 improves cardiac function in heart failure rats

**DOI:** 10.1590/1414-431X20165273

**Published:** 2016-05-31

**Authors:** Y. Lv, B. Liu, H.P. Wang, L. Zhang

**Affiliations:** 1 Department of Histology and Embryology, Hebei Medical University, Shijiazhuang, Hebei, China; 2 Department of Pathology, the First Affiliated Hospital of Hebei North University, Zhangjiakou, Hebei, China; 3 Department of Histology and Embryology, Hebei North University, Zhangjiakou, Hebei, China

**Keywords:** Bone marrow mesenchymal stem cell, Transforming growth factor beta1, Cardiomyocytes, Differentiation, Intramyocardial implantation

## Abstract

The present study tested the hypotheses that *i*) transforming growth factor beta 1 (TGF-β1) enhances differentiation of rat bone marrow mesenchymal stem cells (MSCs) towards the cardiomyogenic phenotype and *ii*) intramyocardial implantation of the TGF-β1-treated MSCs improves cardiac function in heart failure rats. MSCs were treated with different concentrations of TGF-β1 for 72 h, and then morphological characteristics, surface antigens and mRNA expression of several transcription factors were assessed. Intramyocardial implantation of these TGF-β1-treated MSCs to infarcted heart was also investigated. MSCs were initially spindle-shaped with irregular processes. On day 28 after TGF-β1 treatment, MSCs showed fusiform shape, orientating parallel with one another, and were connected with adjoining cells forming myotube-like structures. Immunofluorescence revealed the expression of cardiomyocyte-specific proteins, α-sarcomeric actin and troponin T, in these cells. The mRNA expression of *GATA4* and *Nkx2.5* genes was slightly increased on day 7, enhanced on day 14 and decreased on day 28 while *α-MHC* gene was not expressed on day 7, but expressed slightly on day 14 and enhanced on day 28. Transmission electron microscopy showed that the induced cells had myofilaments, z line-like substances, desmosomes, and gap junctions, in contrast with control cells. Furthermore, intramyocardial implantation of TGF-β1-treated MSCs to infarcted heart reduced scar area and increased the number of muscle cells. This structure regeneration was concomitant with the improvement of cardiac function, evidenced by decreased left ventricular end-diastolic pressure, increased left ventricular systolic pressure and increased maximal positive pressure development rate. Taken together, these results indicate that intramyocardial implantation of differentiated MSCs enhanced by TGF-β1 improved cardiac function in heart failure rats.

## Introduction

Acute myocardial infarction (MI) is the most important manifestation of ischemic heart disease and is one of the leading causes of major morbidity and mortality in the modern world. Recently, with the emergence of myocardial tissue engineering, the delivery of *ex vivo* bone mesenchymal stem cells (MSCs) to the infarcted heart has been successfully performed (1,2).

MSCs are multipotent progenitor cells which can easily be purified and amplified (3,4). Recent studies have shown that MSCs can differentiate into cardiomyocytes (CMCs) or cardiomyocyte-like cells (CLCs) *in vivo* and *in vitro* (5,6). 5-azacytidine is a classic inducer that enhances differentiation of MSCs into CMCs by random demethylation. However, it has been demonstrated that 5-azacytidine is toxic and its differentiation ratio is very low (7).

Transforming growth factor beta 1 (TGF-β1) is a pleiotropic cytokine with many and complex effects in cell and tissue physiology. It is a multifunctional cytokine involved in the differentiation, growth, and survival of a variety of cells (8). In the present study, using different concentrations of TGF-β1 to treat cultured rat bone marrow mesenchymal stem cells (rBMSCs), we first identified the optimal concentration and efficiency of TGF-β1. We employed confocal and electron microscopy, immunofluorescence, and relative quantitative RT-PCR to confirm the enhancing effect of TGF-β1 on cardiogenic differentiation of rBMSCs. Then, we implanted the TGF-β1-treated rBMSCs to the rat infarcted heart to test the improving effect of rBMSCs on cardiac function.

## Material and Methods

### Animals

Ten 3-week-old male Sprague-Dawley rats (35–45 g) for the experiment of cell isolation, and fifty 8-week-old male Sprague-Dawley rats (180–220 g) for the experiment of myocardial infarction model were obtained from Experimental Animal Center of Hebei Medical University. Rats were kept in plastic cages under conditions of controlled temperature (18–21°C) and humidity (55±5%) with a 12/12 h light/dark cycle. The animal experiments were performed in accordance with protocols approved by the Institutional Animal Care and Use Committee of Hebei Medical University.

### Isolation and culture of rBMSCs

Bone marrow was separated from the femur and tibial bones of rats following cervical dislocation. The marrows were collected and diluted with 5 mL of Iscove's modified Dulbecco's medium-low glucose (IMDM-LG; Gibco BRL, USA), supplemented with 15% fetal bovine serum (Gibco), 100 U/mL penicillin, 100 µg/mL streptomycin at 37°C in a humidified atmosphere of 5% CO_2._ After 3 days, non-adherent hematopoietic cells were discarded, and the adherent cells were washed twice with PBS. The culture medium was replenished every 3 days. When the density of the cell colonies reached approximately 90% confluence, the cells were trypsinized with 0.25% trypsin (Amresco, USA) and transferred to fresh flasks at a ratio of 1:2. Using cell markers for MSCs, the cultured cells were identified by flow cytometry.

### rBMSCs induction and differentiation

The 2nd-generation cells were co-incubated with different concentrations of TGF-β1 for 72 h (0, 2, 5, 10, and 15 ng/mL). After cells were washed three times with PBS, and the medium was replaced by pure medium, without any inducers. The medium was replaced every 3 days for 4 weeks after TGF-β1 treatment, and then cells were prepared for the succeeding experiments.

### Immunofluorescence staining for CMCs specific proteins

To identify whether TGF-β1 treated rBMSCs differentiated into CMCs, immunofluorescence staining for the CMC proteins, α-sarcomeric actin and troponin T (cTnT), was performed. The cells were transferred to sterile glass cover slips, followed by 4% formaldehyde for 15 min. After blocking with 2% bovine serum albumin (BSA) for 1 h, the cells were incubated with both monoclonal rabbit anti-α-sarcomeric actin primary antibody (1:50, Abcam, England) and polyclonal goat anti-troponin T primary antibody (1:50, Abcam) at 4°C for 24 h. Then, cells were stained with rhodamine-conjugated anti-rabbit secondary antibody and FITC-conjugated anti-goat secondary antibody for 60 min and washed with PBS three times. Negative controls were also employed to offset the disturbance of the primary or secondary antibody. The results were observed and recorded by fluorescence microscopy (Leica TCS-ST2 Instrument, Japan).

### Transmission electron microscopy

After 4 weeks, cells were harvested and fixed with 3% glutaraldehyde and 1% osmium tetroxide, then embedded in epoxy resin. Ultra-thin sections were cut horizontally and double-stained with uranyl acetate and lead citrate. The cellular ultrastructure was observed using a JEM-2000EX transmission electron microscope (TEM) (Japan).

### Analysis of rBMSCs-cardiac differentiation by semi quantitative RT-PCR

The transcription factors *GATA-4*, *Nkx2.5*, *α-MHC* in TGF-β1 treated cells were assessed by semi quantitative RT-PCR on day 7, 14, or 28. Total RNA was extracted using RNAfast200 Kit (Fastagen, China) according to the manufacturer's protocol. RNA was then reverse transcribed into cDNA using M-MLV Rtase cDNA Synthesis Kit (Invitrogen, China). The endogenous 'house-keeping' gene (*GAPDH*) was used to evaluate the efficiency of reverse transcription. PCR was performed using 2×Taq PCR Master Mix (Tiangen, China). Cycle conditions were as follows: 94°C for 3 min followed by 30 cycles of 94°C denaturation for 30 s, 58.4°C annealing for 30 s, 72°C extension for 1 min, with a final incubation at 7°C for 5 min. The PCR products were analyzed by electrophoresis on 2% agarose gel. The sequences of the primers are shown in Table 1.

**Table:**
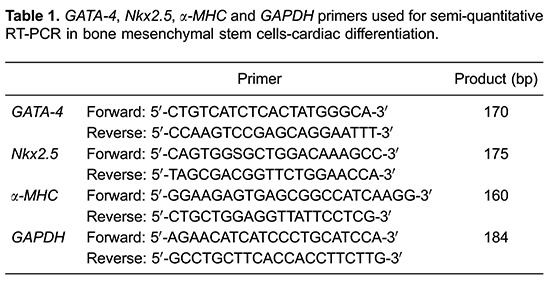

### Intramyocardial implantation of MSCs treated with 5 ng/mL of TGF-β1

MI was conducted by ligation of the left anterior descending coronary artery. After the rats were anesthetized with 3.5% chloral hydrate (35 mg/kg), the tracheas were inserted into a casing, which was connected to the ventilator. The mechanical ventilation parameters were adjusted (expiration/inspiration=1.5:1, respiratory frequency 70 bpm). Then, a left thoracotomy through the fourth intercostal space and the left anterior descending artery ligation were conducted. The hearts were inserted back into the chest rapidly for heart beat recovery, for 1 min. When the breath of the rats was even, the hearts were quickly taken out again and the cell suspension (1×10^6^/100 µL) was injected into the junction of the infarction area and normal myocardial area.

The rats were randomly divided into 3 groups: sham operation group (sham, n=10), acute myocardial infarction with 100 µL IMDM-LG injection (MI, n=10), and acute myocardial infarction with exogenous transplantation of rBMSCs treated with 5 ng/mL TGF-β1 (TGF-β1, n=10). In the TGF-β1group, MSCs were first treated with TGF-β1 for 72 h, then new media was added (excluding inductive substance) and cells were cultured for 1 h. After that, the cell suspension was injected into the junction of infarction area and normal myocardial area. After surgery, ketoprofen (5 mg/kg, *sc*) was given for 3 days for postoperative pain relief. The overall mortality rate of heart failure rats during the entire experimental period (up to 4 weeks after MI) was 30 to 40%. The majority of deaths occurred on the day of, or the day after, the MI surgery, probably due to acute pump failure or lethal arrhythmias. Cardiovascular hemodynamics were tested 4 weeks after surgery. The rats were anesthetized with sodium pentobarbital and the tracheas were intubated. A polyethylene catheter was introduced into the right carotid artery. Then, the catheter was connected to a pressure transducer (TP-200T; Nihon Kohden, Japan) and the heart rate was measured. After this procedure, a catheter was inserted into the left ventricular via right common carotid artery, from which left ventricular systolic pressure (LVSP), left ventricular end-diastolic pressure (LVEDP), as well as maximal positive and negative pressure development rates (+dP/dt and -dP/dt) were measured. At the end of the experiment, heart samples were harvested for Masson staining (Masson Trichrome Stain Kit, Leagene Biotechnology, China). Five sections were selected for each specimen, and four views were taken for each section. The Image-Pro Plus 6.0 software (Olympus, Japan) was used to calculate the myocardial collagen volume fraction (CVF).

### Statistical analysis

Data are reported as means±SD. One-way ANOVA was used to analyze the differences in mRNA expression of the transcription factors, cardiac hemodynamics and myocardial CVF. Other comparisons were performed with the chi-square test. Statistical analysis was performed using the SPSS software (USA). Differences were considered to be statistically significant at P<0.05 with a 95% confidence interval.

## Results

### Morphological alteration of rBMSCs

**Figure 1:**
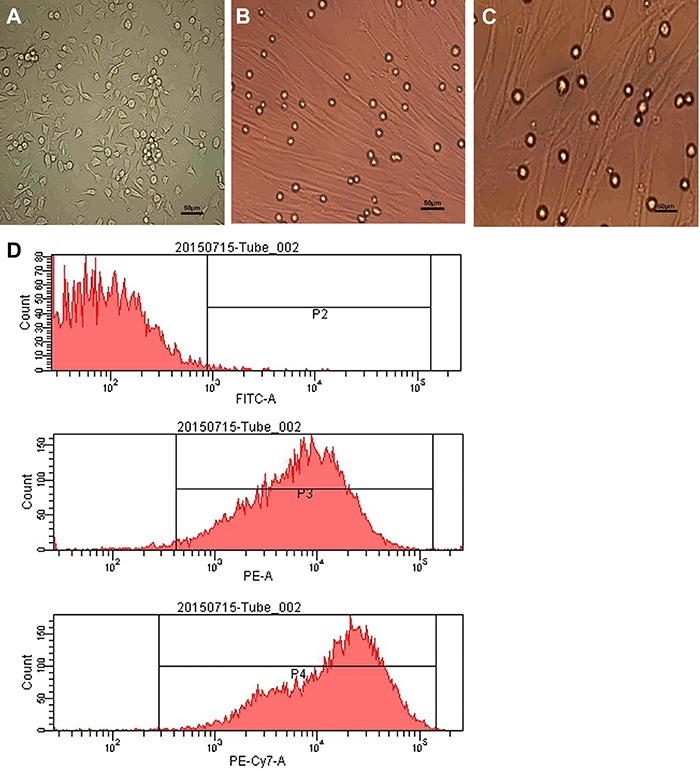
Morphologic change of rat bone marrow mesenchymal stem cells (rBMSCs). *A*, rBMSCs cultured on day 7; *B*, rBMSCs treated with 5 ng/mL TGF-β1 on week 4; *C*, rBMSCs treated with 10 ng/mL TGF-β1 on week 4; *D*, flow cytometry identification of rBMSCs. Magnification bar: 50 μm.

After 12 h of primary culture, rBMSCs began to adhere to the culture bottle. Three days later, rBMSCs were circular or short spindle-shaped with one nucleus. These cells began to proliferate on day 7 and gradually grew to form small colonies (Figure 1A). After being subcultured, the cells were either polygonal or long and spindle-shaped.

The flow cytometry test revealed that CD29, CD90 (fibers connecting receptor) were positively expressed, while CD45 (hematopoietic stem cell marker) was negatively or weakly expressed in the isolated rBMSCs, which was indicative of mesenchymal stem cell (Figure 1D).

After the 2nd-generation rBMSCs were induced by different concentrations of TGF-β1 for 72 h, the morphological differentiation from rBMSCs to CLCs was initiated. The differentiated cells appeared single or in group. Cells were spindle-shaped or branched, with one or two round nucleus located in the center. After 10 days, the rBMSCs were differently developed in each group. The 2 or 15 ng/mL TGF-β1 treatment induced spindle-shaped rBMSCs with irregular processes, which was similar to the changes in the control group. In contrast, the 5 ng/mL TGF-β1 treatment induced rBMSCs to be in complete contact with adjoining cells, and to have a fusiform shape, with a parallel orientation with one another, thereby forming myotube-like structures on week 4 (Figure 1B). The shape of rBMSCs treated with 10 ng/mL TGF-β1 was similar to those treated with 5 ng/mL TGF-β1, but the amount of cells was relatively lower (Figure 1C).

### CMC-specific protein expression during rBMSCs differentiation

**Figure 2:**
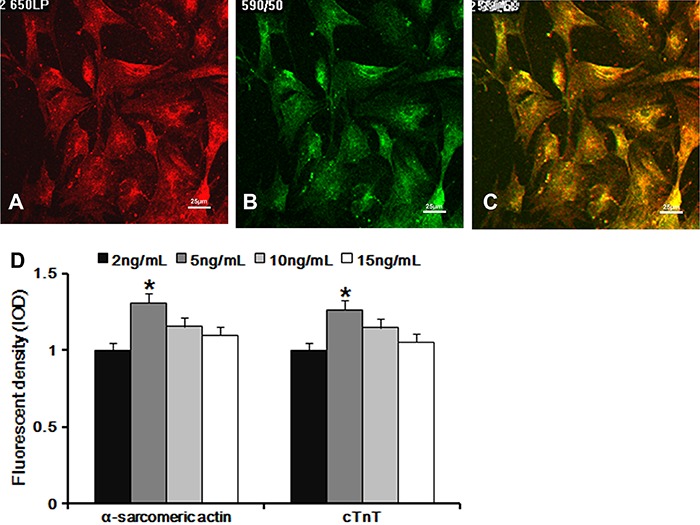
Laser scanning confocal microscopy results used to investigate double labeling of α-sarcomeric actin and cTnT proteins in rat bone marrow mesenchymal stem cells (rBMSCs) treated with 5 ng/mL TGF-β1, evaluated by fluorescence. *A*, Red light indicating α-sarcomeric actin; *B*, green light indicating cTnT; *C*, the presence of both proteins was indicated by yellow light; *D*, bar graphs showing the double labeling of α-sarcomeric actin and cTnT of rBMSCs treated with different concentrations of TGF-β1. Magnification bar: 25 μm. Data are reported as means±SD. *P<0.05 *vs* 2 ng/mL group (one-way ANOVA).

On week 4, most of the cells in TGF-β1 treated group presented both α-sarcomeric actin (red) and cTnT proteins (green), differently from the control group. Analysis of the fluorescence intensity revealed that the expression of α-sarcomeric actin and cTnT in cells treated with 5 ng/mL TGF-β1 (Figure 2A-C) were noticeably higher than that of the other groups (Figure 2D).

### Ultrastructural characterization of differentiated cells

**Figure 3:**
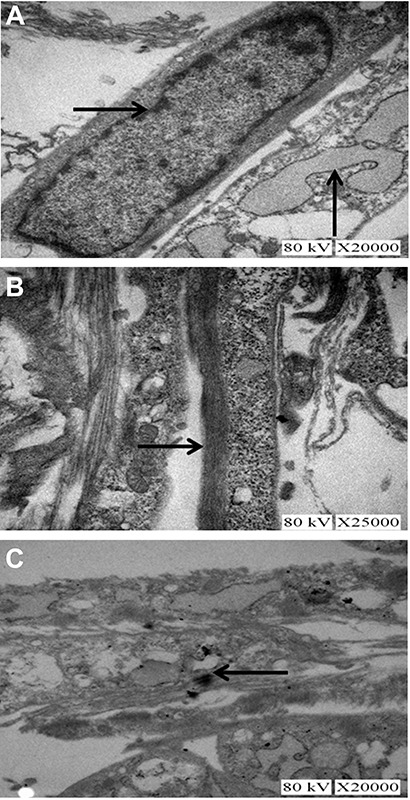
Transforming growth factor beta induced rat bone marrow mesenchymal stem cells evaluated by a transmission electron microscope. *A*, Abundant organelles, such as rough endoplasmic reticulum and mitochondria (vertical arrow), can be seen in the cytoplasm, with an oval nucleus (horizontal arrow) located in the center of the cells, ×20,000. *B*, Myofilaments (horizontal arrow) were found in parallel in the cytoplasm, with light and dark transverse striation, ×25,000. *C*, Gap junction (horizontal arrow) between cells, ×20,000.

Under TEM, the cells induced by TGF-β1 showed abundant organelles with an oval nucleus located in the center of the cells (Figure 3A, horizontal arrow). These organelles contained a large number of rough endoplasmic reticulum, mitochondria (see Figure 3A, vertical arrow), glycogen and ribosomes. Myofilaments were found to be in parallel in the cytoplasm (Figure 3B, horizontal arrow) with light and dark transverse striation. Gap junctions between the cells (Figure 3C, arrow), which are characteristic of CMCs, were also clearly visible. These are properties that indicate myofilament installation during CMCs development. Ultrastructure observation results in rBMSCs treated with 5 ng/mL TGF-β1 were more typical of CMCs than that of other treatment groups. In contrast, no myofilaments and light and dark transverse striations were detected in the control group.

### mRNA expression of transcription factors during rBMSCs-cardiac differentiation

**Figure 4:**
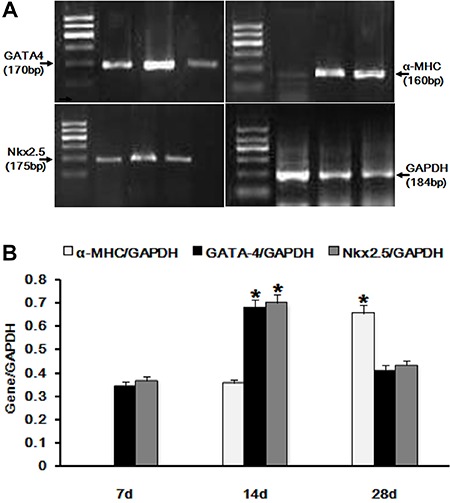
Expression of mRNA for *GATA-4*, *Nkx2.5*, *α-MHC* in rat bone marrow mesenchymal stem cells treated with 5 ng/mL TGF-β1. *A*, Expression of mRNA by electrophoresis on 2% agarose gel. *B*, Bar graphs showing the semi-quantitative expression of mRNA for *GATA-4*, *Nkx2.5* and *α-MHC*, for the 28-day experiment. Data are reported as means±SD. *P<0.05 *vs* 7d (one-way ANOVA).

mRNA expressions of *GATA-4*, *α-MHC* and *Nkx2.5* were measured by semi-quantitative RT-PCR. The *GATA4* and *Nkx2.5* genes were weakly expressed on day 7, enhanced on day 14, and decreased on day 28; *α-MHC* gene was not presented on day 7, weakly expressed on day 14, and enhanced on day 28 (Figure 4, A and B). The expression of these genes in cells treated with 5 ng/mL TGF-β1 was higher than that of cells treated with other doses of TGF-β1, at each time point (Table 2).

**Table:**
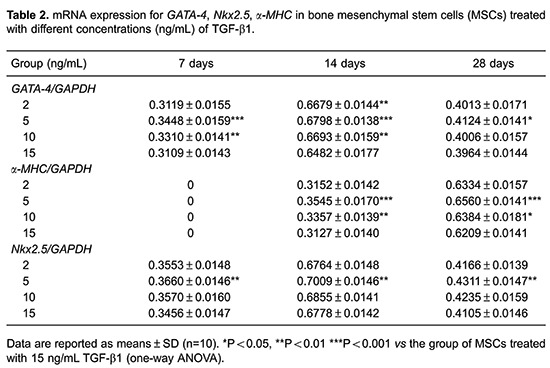

### Effects on cardiac function by the implantation of MSCs treated with 5 ng/mL of TGF-β1

Cardiac hemodynamics was tested on week 4 after intramyocardial transplantation of MSCs treated with 5 ng/mL TGF-β1. Compared with sham group, the MI group without MSCs implantation showed a decreased LVSP and ±dp/dt max, and an increased LVEDP. In contrast, myocardial implantation of MSCs in infarcted heart partially reversed these changes in cardiac hemodynamics (Table 3).

**Table:**
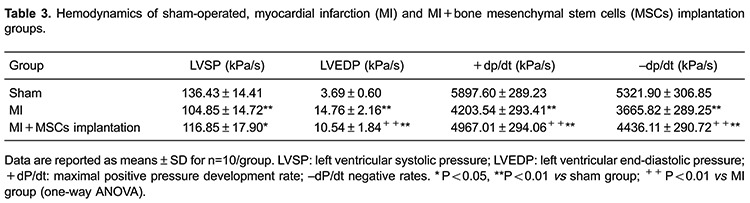

**Figure 5:**
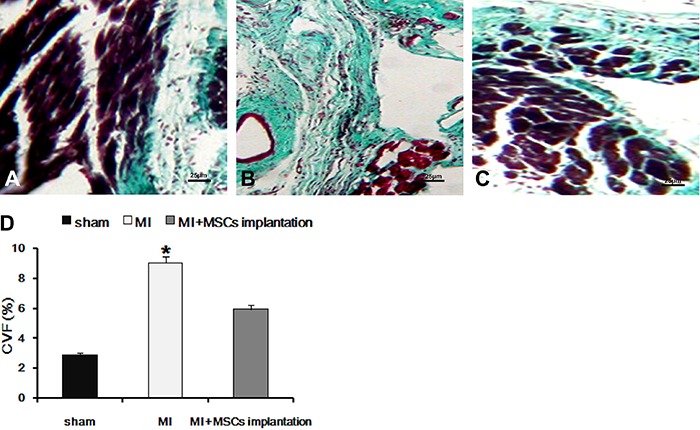
Masson staining results. *A*, sham operation group; *B*, after myocardial infarction, a scar area dyed green or blue appears in the control group; *C*, muscle cells dyed red can be observed between collagen fibers in the group that received myocardial implantation of mesenchymal stem cells (MSCs) in the infarcted heart; *D*, myocardial collagen volume fraction (CVF) of different groups. MI: myocardial infarction. Magnification bar: 25 μm. Data are reported as means±SD. *P<0.05 *vs* sham operation group (one-way ANOVA).

Furthermore, Masson staining showed that, differently from sham group (Figure 5A), a large scar area stained green or blue was present in the MI heart without MSCs implantation (Figure 5B). In contrast, many muscle cells with red staining were present between collagen fibers in MSCs treated heart (Figure 5C). MI group without MSCs implantation showed an increased CVF compared with the sham group (P<0.05). In contrast, myocardial implantation of MSCs in infarcted heart partially reversed these changes (Figure 5D). The results indicate that intramyocardial implantation of TGF-β1-treated rBMSCs to infarcted heart reduced the scar area and increased muscle cells.

## Discussion

The major findings of the present study are *i*) TGF-β1 treatment induced an enhanced differentiation of rBMSCs towards cardiogenic cells, and the optimal concentration of TGF-β1 was found to be of 5 ng/mL; *ii*) intramyocardial implantation of the TGF-β1-treated rBMSCs to the infarcted heart reduced scar area, increased muscle cells and improved cardiac function. Taken together, these results indicate that TGF-β1 is an efficient inducer for rBMSCs differentiation, and intramyocardial implantation of TGF-β1-treated rBMSCs to the infarcted heart may represent a potential therapeutic strategy for the treatment of ischemic heart disease.

MSCs are non-specialized cells with the ability of self-renewal and multiple differentiation potential. They can be easily isolated from bone marrow, adapted to *ex vivo* expansion, and differentiated into multilineage cells both *in vitro* and *in vivo*, if ethical concerns and immunological rejection were not accounted for (9). *In vivo* and *in vitro* studies show that the source of stem cells is crucial for successful implantation (10). MSCs harvested from young rodents demonstrate significantly increased cellular proliferation, greater resistance to hypoxic conditions, and improved differentiation compared with MSCs obtained from older rodents. Thus, in the present study, 3-week-old rats were chosen to achieve the best possible results.

It has been demonstrated that MSCs can differentiate into cardiomyocytes *in vivo* and *in vitro* (11,12). 5-azacytidine is a classic inducer of MSCs for their differentiation towards cardiomyocytes. However, 5-azacytidine treatment could not induce differentiation of rBMSCs in an expected cardiomyogenic way (13), with a differentiation rate of no more than 30% (14,15). Furthermore, the efficient concentration of 5-azacytidine for cardiomyogenic differentiation is very high (10 mM) which produces toxicity and side effects (16). Therefore, it is necessary to find a new inducer to safely increase the MSCs differentiation rate.

TGF-β1 is a well-documented potent chondrogenic factor. Recent studies have shown that TGF-β1 can improve the cardiogenic differentiation of MSCs *in vitro* (17). For an angiogenesis effect, TGF-β1 displays a biphasic role. Low concentrations of TGF-β1 synergistically enhance, whereas high concentrations decrease the vascular invasion of cultured endothelial cells induced by angiogenic factors (18). Therefore, different concentrations of TGF-β1 may play different roles in cell proliferation and differentiation, bone formation, angiogenesis, cell cycle progression and cellular migration. In the present study, four different concentrations of TGF-β1 were tested to find the optimal concentration that would efficiently differentiate rBMSCs into CMCs or CLCs.

To provide a reliable comparison of rBMSCs differentiation potency towards the cardiomyogenic phenotype using different concentrations of TGF-β1, *in vitro* experiments were contrasted by confocal and electron microscopy, immunofluorescence and relative quantitative RT-PCR. Myocardial related markers, such as α-sarcomeric actin, cTnT, *Nkx2.5*, *α-MHC* and *GATA-4* were detected in the experiments. cTnT has been demonstrated to be exclusively present in cardiac muscle, and is a proven diagnostic and risk stratification biomarker in patients with acute coronary syndromes. *Nkx2.5* and *GATA-4* are required for specification of the cardiac muscle phenotype (19). *Nkx2.5* regulates the transcription of several cardiac genes including α-sarcomeric actin and *GATA-4* (20). Moreover, cell gap junction is an important feature of the cardiomyocyte that provides the capacity of the cells to grow and migrate (21). In the present study, we found that TGF-β1 treated rBMSCs showed an increased expression of cardiac-specific markers including cTnT, *GATA-4* and *Nkx2.5*. Gap junctions were also found with transmission electron microscopy. These results suggest that TGF-β1 is a potent inducer of BMSCs differentiation into cardiomyocyte-like cells. Furthermore, we found that proteins and mRNA expression levels were different in cells treated with different concentration of TGF-β1. The expression levels in cells treated with 5 ng/mL of TGF-β1 were significantly higher than that of the other groups at each time point. High doses of TGF-β1 may have a cytotoxic effect that lead to programmed cell death and senescence, which could result in a low differentiated rate. Our results indicated that 5 ng/mL of TGF-β1 may be the optimal concentration for cardiomyogenic differentiation.

MSCs have been shown to be a promising therapeutic strategy in preclinical studies (22,23). A recent study showed that MSC could improve myocardial function and promote myofibroblasts to congregate in the infarcted region via activation of the TGF-β1-Smad2 signaling pathway in an MI model (24). However, limited clinical success was observed, mainly due to poor MSC survival and harsh microenvironment conditions. Li et al. (25) reported that intramyocardial implantation of TGF-β-preprogrammed bone marrow stem cells, but not untreated cells, resulted in a regeneration of myocardium in the left ventricular anterior wall and an increased left ventricular percent fraction shortening, in coronary ligation mice. The present study confirms and extends that study by showing that intramyocardial implantation of TGF-β1-treated rBMSCs to infarcted rat heart reduced scar area and increased muscle cells. This structure regeneration was paralleled with improvement of cardiac function by showing increased LVSP and ±dp/dt max, and deceased LVEDP.

The therapeutic effect of MSCs is not a result of the cells’ action, but it is a process coordinated with the local microenvironment where the MSCs are implanted (26). Understanding such interaction should be helpful in choosing an optimal dose and time points for MSC implantation, and in predicting the range in which MSCs should be effective. Further studies need to be performed to explore these related issues and the precise mechanism underlying the effects of TGF-β1 treated MSCs implantation on cardiac function.

In conclusion, the present study demonstrated that TGF-β1 promoted cardiomyogenic differentiation of rBMSCs, and that the implantation of these cells can improve the function of the injured myocardial tissue, which may represent a potential therapeutic strategy for the treatment of ischemic heart disease.
